# Surgical treatment strategy for esophagogastric junction cancers based on the tumor diameter

**DOI:** 10.1186/s12893-019-0614-5

**Published:** 2019-10-24

**Authors:** Isamu Hoshino, Hisashi Gunji, Fumitaka Ishige, Yosuke Iwatate, Nobuhiro Takiguchi, Atsushi Ikeda, Hiroaki Soda, Toru Tonooka, Nami Sato, Kenji Kawahara, Yoshihiro Nabeya

**Affiliations:** 1 Division of Gastroenterological Surgery, Chiba Cancer Center, 666-2 Nitonacho, Chuo-ku, Chiba, 260-8717 Japan; 2 Division of Hepatobiliary and Pancreatic Surgery, Chiba Cancer Center, 666-2 Nitonacho, Chuo-ku, Chiba, 260-8717 Japan

**Keywords:** Esophagogastric junction cancer, Surgical treatment, Tumor diameter, Lymph nodal dissection

## Abstract

**Background:**

The number of patients with esophagogastric junction (EGJ) cancers has tended to increase. However, no clear consensus on the optimum treatment policy has yet been reached.

**Methods:**

This study included patients diagnosed with adenocarcinoma of Sievert type II in whom resection was performed in our hospital. We performed a clinicopathological examination, and patients were divided into two groups by the tumor size: L group, tumor size ≥4 cm; and S group, tumor size < 4 cm. The clinical factors, such as nodal dissection and recurrence pattern, were then analyzed.

**Results:**

A total of 48 patients were diagnosed with ECJ cancers. The average tumor size was 55.1 mm, and 32 cases (66.7%) had tumors ≥4 cm. Metastasis to the mediastinum was noted in 4 cases (12.5%) in the L group but none in the S group. Recurrence in the upper or middle mediastinum lymph nodes was noted in 3 cases (9.4%) in the L group. The 5-year overall survival rates were 49.7 and 83.9% in the L and S groups, respectively.

**Conclusions:**

As the tumor grows large, it is difficult to accurately judge EGJ on the image, and as a result it is difficult to understand the exact esophageal invasion distance of the tumor. Therefore, lymph node dissection including the upper mediastinum is considered vital, regardless of the degree of esophageal invasion.

## Background

The rate of adenocarcinomas at the esophagogastric junction (EGJ) has been increasing rapidly for the past 20 years in certain areas, mainly in Europe and the United States. In contrast, in Japan and other Asian countries, including Korea and China, squamous cell carcinoma accounts for 90% of esophageal cancer cases [1]. Over the past 20 years, according to the Comprehensive Registry of Esophageal Cancer in Japan, the frequency of EGJ cancer in Japan has gradually increased from 5.5 to 6.9% [2]. In addition, the proportion of adenocarcinoma as the histological type among esophageal carcinomas is still increasing, rising from 3.1 to 5.3% in the same period. The *Helicobacter pylori* infection rate has decreased significantly in many Asian countries, including Japan, thanks to eradication efforts [3, 4]. However, *H. pylori* infection has been shown to inhibit the development of Barrett esophagus and Barrett esophageal cancer [5]. Of note, the occurrence of Barrett esophagus is associated with gastroesophageal reflux disease (GERD) [6–8]. Furthermore, dietary habits in Asian countries are becoming increasingly Westernized, which will eventually lead to Westernization of the body shape and an associated increase in the risk of EGJ cancer.

Regarding treatment, surgery is considered the mainstay management method for EGJ cancer, regardless of the peri-operative adjuvant therapies performed. However, the ideal surgical operation for EGJ cancers varies markedly depending on the location site. In addition, there is no consensus concerning the operation, so surgical techniques at present differ among facilities, regions, and countries. EGJ carcinoma is classified into Sievert type I, type II, or type III depending on the position of the center of the tumor. In general, Sievert type I is considered to resemble esophageal cancer, while Sievert type III is often treated with a similar surgical procedure to gastric cancer. However, the appropriate surgical approach for EGJ cancers of Sievert type II, which are categorized as true junctional carcinomas, has yet to be conclusively decided.

In Japan, the Japanese Gastric Cancer Association (JGCA) and Japan Esophageal Society (JES) jointly established a working group and performed a retrospective study of optimal lymph node dissection of EGJ cancers [9]. In that study, it was judged that the center of the tumor could not be accurately identified anatomically when the tumor was large, so the study was limited to cases with a long diameter of < 4 cm. However, clinical studies on EGJ cancers performed thus far suggest that the median tumor size in such cancers is about 5 to 7 cm; tumors ≥4 cm in size therefore account for the majority of lesions, so their management needs to be addressed [10, 11]. However in the case of a large tumor size, it is difficult to accurately determine the position of the EGJ.

In the present study, we conducted a clinicopathological examination of EGJ cancers treated in our department and examined the ideal approach to lymph node dissection by dividing the tumor diameter into ≥4 cm and < 4 cm.

## Methods

### Patients

Forty-eight patients with EGJ cancers who underwent resection of the primary tumor from January 2006 to December 2017 were included in this study. EGJ cancers in this analysis were determined as tumors with an epicenter within 2 cm proximal or distal to the anatomical EGJ, according to the definition advocated by the JGCA [12] and JES [13, 14]. Tumor diameter was measured before formalin fixation after specimen removal.

Tumors diagnosed histologically as adenocarcinoma were chosen, and other histological types of tumor were excluded. Pathologic T, N, and M stages were based on the International Union Against Cancer tumor-node-metastasis (UICC) TNM staging system for EGJ cancer, 7th edition. Lymph node station numbers were determined according to the uniform definition established by the JGCA and JES. Over 5 years after surgery, CT scans for follow-up recurrence were performed at least once every 6 months. We also analyzed the correlation with histopathological factors after dividing the patients with a tumor diameter ≥ 4 cm into the L group and those with a tumor diameter < 4 cm into the S group.

Approval to conduct this study was obtained from the institutional ethics review board of the Chiba Cancer Center (H29–262).

### Statistical analyses

All statistical calculations were performed using the JMP Pro 13 software program, (SAS Institute, Cary, NC, USA). The overall survival (OS) and recurrence-free survival (RFS) rates were calculated from the surgery date to the date of death due to any cause and first recurrence or death due to any cause, whichever came earlier, respectively. Survival curves were calculated using the Kaplan-Meier method. *P* values less than 0.05 were considered to indicate a statistically significant difference in all analyses.

## Results

### Characteristics of the patients

There were 2225 and 331 cases in which curative resection was performed for gastric and esophageal cancer, respectively, in our hospital between January 2010 and December 2017. Among them, 48 cases underwent resection surgery for EGJ cancers (adenocarcinoma) (Table 1). There were 42 men and 6 women, with an average age of 66.1 years old and an average tumor diameter of 55.1 mm, which was relatively large. The mean tumor epicenter was + 4.8 mm on the gastric side.

**Table 1:** Characteristics of 48 patients with EGJ cancers

Characteristics	No. of Patients (%)
Sex	
Male	42 (87.8)
Female	6 (12.2)
Age	66.1 (41–87)
Tumor size (mm)	55.1 (10–147)
Center of the tumor from EGJ (mm)	
Median (range)	+ 4.8 (−12.5 to + 20)
pT category	
T1a or b	11 (22.9)
T2	18 (37.5)
T3 or T4	19 (39.6)
pN category	
N0	18 (37.5)
N1	15 (31.3)
N2	10 (20.8)
N3	5 (10.4)
Number of metastatic lymph nodes	
Median (range)	5.3 (0–39)
lymphovascular invasion	
Negative	7 (14.6)
Positive	41 (85.4)
Approach	
Right transthoracic	4 (8.3)
Left transthoracic	17 (35.4)
Transhiatal	27 (56.3)
Type of esophageal resection	
Total/subtotal esophagectomy	5 (10.4)
Lower/abdominal esophagectomy	43 (89.6)
Type of gastric resection	
Total gastrectomy	24 (50.0)
Proximal gastrectomy	24 (50.0)

**Fig. 1:**
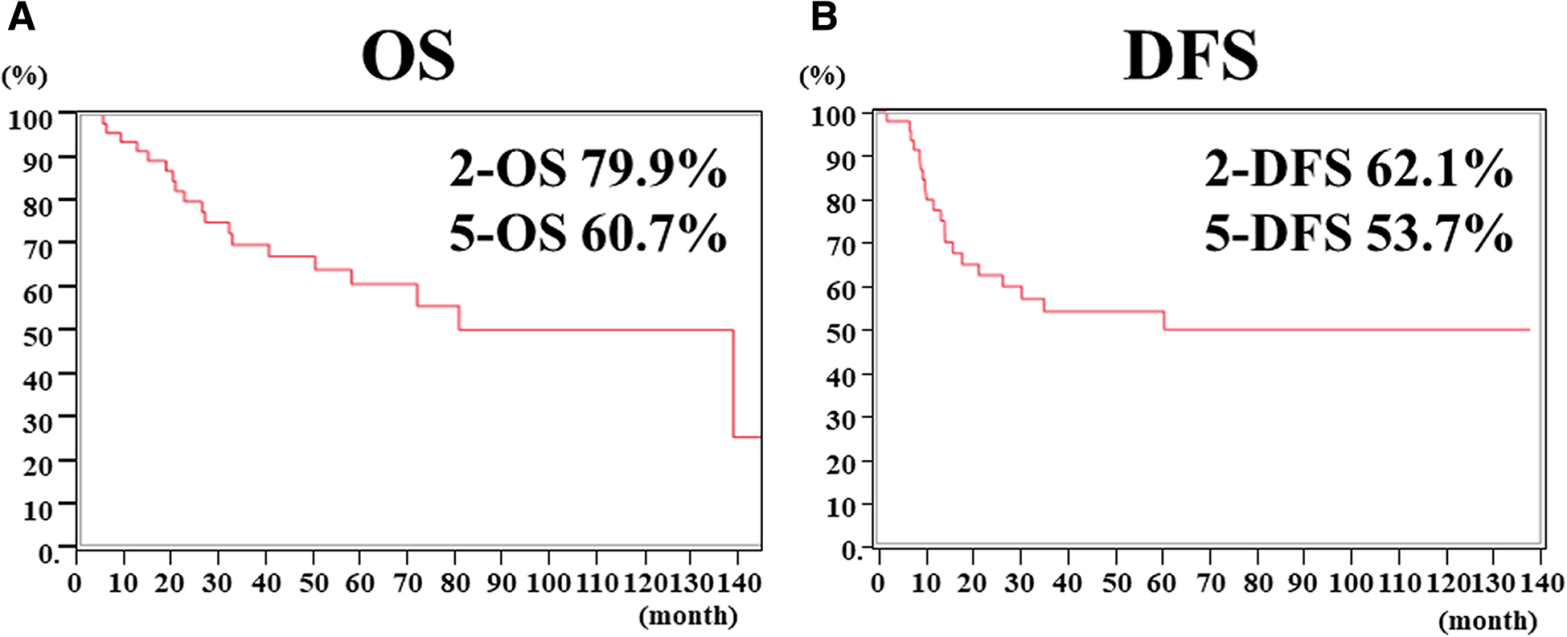
**a** Overall survival (OS) curves of EGJ cancers, **b** Disease free survival (DFS) curves of EGJ cancers

Among them, 11 cases (22.9%) were classified as pT1a or pT1b, 18 cases (37.5%) as T2, and 19 cases (39.6%) as T3 or T4. Regarding lymph node metastasis, 18 cases (37.5%) were negative, 15 cases (31.3%) were N1, 10 cases (20.8%) were N2, and 5 cases (10.4%) were N3. The mean number of lymph node metastases was 5.3. Lymphovascular invasion was negative in only 7 cases (14.6%), and the remaining 41 cases (85.4%) were positive. The transhiatal approach was the most frequently adopted route for approaching the mediastinum, being performed in 27 cases (55.3%); 17 cases (35.4%) underwent left thoracotomy, and only 4 (8.3%) underwent right thoracotomy. Regarding gastrectomy, 24 cases (50.0%) underwent total gastrectomy, and 24 (50.0%) underwent cardia-side gastrectomy (including stomach partial resection by gastric tube reconstruction). In the analysis of the prognosis, the 5-year OS was 60.7%, and the 5-year disease-free survival was 53.7% (Fig. 1).

### Rate of dissection and metastasis of lymph node at mediastinum

**Fig. 2:**
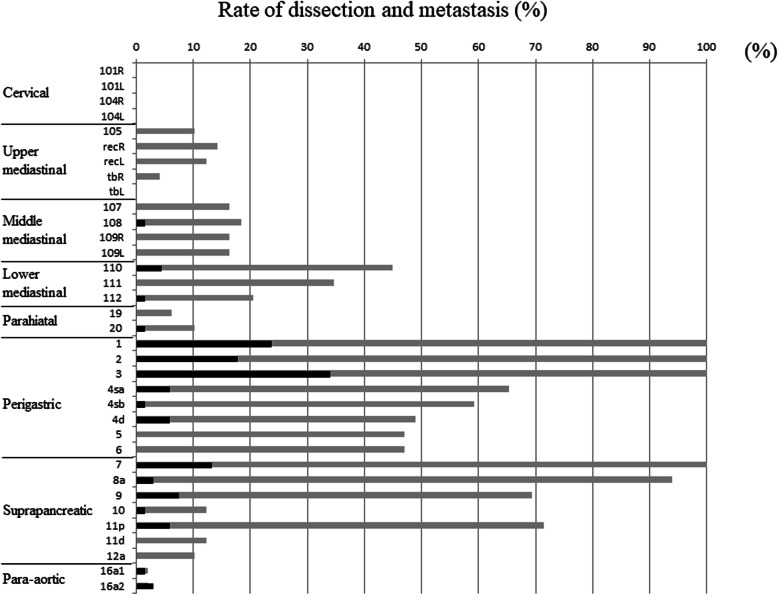
Rates of lymph node dissection and metastatis. The light black color indicates the rates of dissection, and the dark black color indicates the rates of metastasis

The rates of lymph node dissection and metastasis are shown in Fig. 2. In this observational study, no cases showed metastasis to the superior mediastinum. Only one case showed metastasis to node no. 108 (middle paraesophageal lymph node), which is a mediastinal lymph node. In that case, the epicenter was + 8 mm on the gastric side, but the maximum tumor diameter was 147 mm, indicating a huge tumor. In addition, there were 3 cases in which metastasis to the inferior mediastinum was found. In total, only 4 cases (8.3%) had lymph node metastasis to the mediastinum (Table 2).

**Table 2:** Rates of lymph node metastasis. Lymph node metastasis is stratified into L (long) group and S (short) group and evaluated

	Total (*n* = 48)	L group (32)	S group [15]	*P* value
Sex				
Male	42 (87.8%)	29 (87.9)	13 (81.3)	0.643
Female	6 (12.2%)	3 (12.1)	3 (18.7)	
Age	66.1	62.6	68	0.416
pN (+) total	30 (62.5%)	22 (68.8)	7 (43.8)	0.55
pN (+) mediastinum	4 (8.3%)	4 (12.5)	0 (0)	0.29
upper mediastinum	0	0	0	0.85
middle mediastinum	1	1	0	
lower mediastinum	3	3	0	

### Site of lymph node recurrence

Recurrence developed in 16 cases (33.3%) in total (Table 3). Among them, 9 (18.8%) had lymph node metastasis recurrence, and 3 had lymph node recurrence to the mediastinum (1 case of lymph node recurrence to the upper mediastinum and 2 cases of lymph node recurrence to the middle mediastinum). Lymph node recurrence to the lower mediastinum was not observed. Most of the remaining cases of lymph node recurrence outside of the mediastinum were recurrence to the paraaortic lymph node.

**Table 3:** Rates of lymph node recurrence. Lymph node recurrence is stratified into L (long) group and S (short) group and evaluated

	Total (*n* = 48)	L group (32)	S group [15]	*P* value
Recurrence	16 (33.3%)	14 (43.8)	2 (12.5)	0.17
lymph node metastasis	9 (18.8%)	7 (21.9)	2 (12.5)	0.79
upper mediastinum	1	1	0	0.83
middle mediastinum	2	2	0	
lower mediastinum	0	0	0	
others	6	4	2	0.65

### An analysis of the recurrence pattern according to the tumor diameter

**Fig. 3:**
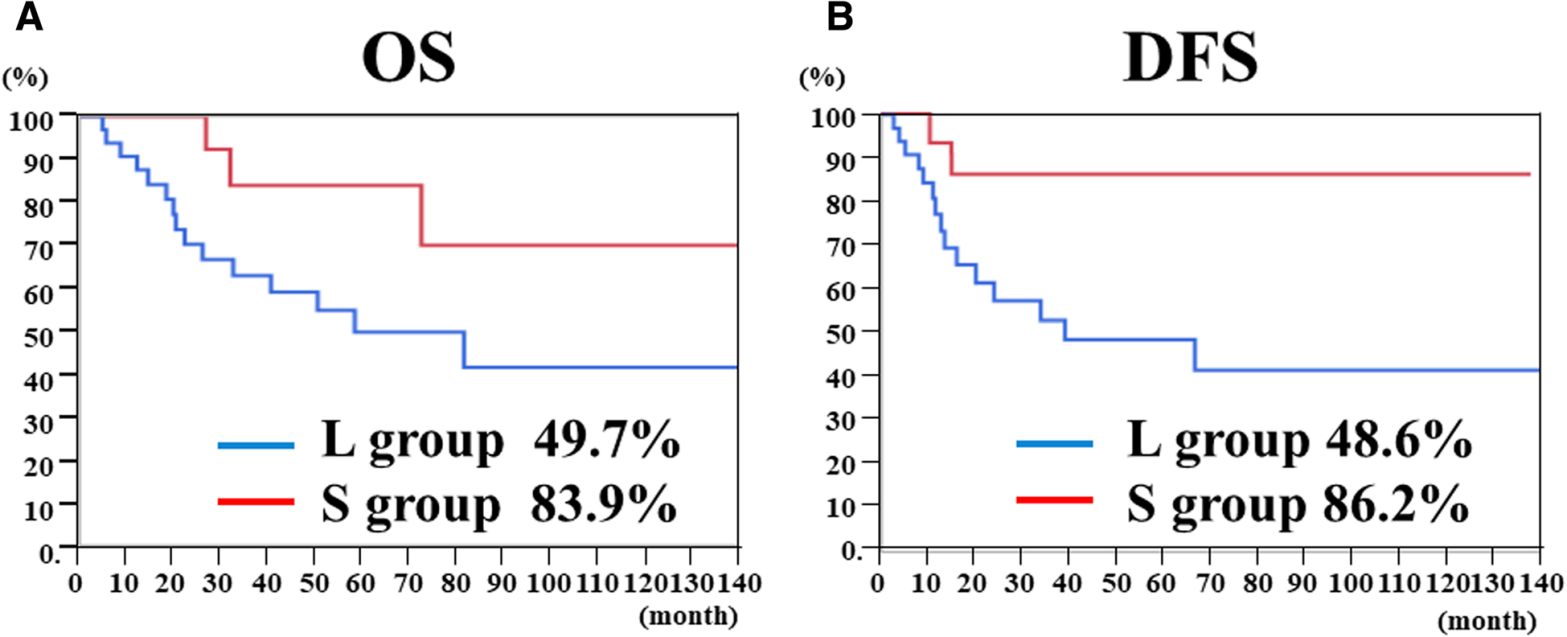
**a** Five years Overall survival (OS) curves of EGJ cancers, **b** Five years Disease free survival (DFS) curves of EGJ cancers. Analysis was done when 5-year survival rate is divided into L (long) group and S (short) group

The 48 cases were divided into 2 groups based on the tumor diameter (L and S groups) and analyzed (Tables 2 and 4). There were no differences in baseline characteristics such as age and gender in each group. The L group included 32 cases (66.7%), while the S group included 16 cases (33.3%). All four cases of lymph node metastasis to the mediastinum were in the L group. In addition, 14 cases (43.8%) showed recurrence in the L group, whereas only 2 cases (12.5%) showed recurrence in the S group. Lymph node recurrence was noted in 7 patients (21.9%) in the L group, 3 of which were recurrence to the mediastinum. Even in S group, lymph node recurrence was observed in 2 cases. However, these lymph node recurrence cases were cases of paraaortic lymph node recurrence, neither of which was a case of lymph node recurrence in mediastinal site. The 5-year OS rates were 50.6 and 80.0% in the L group and S group, respectively (*p* = 0.043) (Fig. 3). The 5-year disease-free survival rates were 48.6 and 86.2% in the L group and S group, respectively (*p* = 0.038) (Fig. 3).

**Table 4:** Baseline characteristics in L group and S group

Characteristics	L group (*n* = 32) (%)	S group (*n* = 16)(%)	*P* value
Sex			
Male	29	13	0.643
Female	3	3	
Age	62.6	68	0.416

## Discussion

A number of different surgical treatments are available for managing EGJ cancer. In recent years, it is thought that surgery under the laparoscopic or thoracoscopic procedures are increasing. With thoracoscopic surgery, which is described as minimal invasive surgery, it is possible to avoid thoracotomy. However, whether or not the invasiveness of thoracoscopic surgery is indeed reduced compared to open surgery remains controversial, and it has not yet been definitively concluded that it leads to a reduction in complications [15–20]. Therefore, criteria to determine the need for thoracic manipulation could be very beneficial to the patient.

A Dutch trial and the JCOG 9502 trial, which is a randomized controlled trial evaluating lymph node dissection of the middle and lower mediastinum for EGJ cancer, were conducted [11, 21]. In the Dutch trial, the outcomes of right thoracotomy versus hysterectomy were compared for Sievert types I and II, with significantly more respiratory-related complications noted in the thoracotomy group than in the transhiatal group (57% vs. 27%, *p* < 0.001). Regarding the long-term results, there were no marked differences in the 5-year survival rate between the thoracotomy group and hysterectomy group for Sievert type II (27% vs. 31%, NS). In the JCOG 9502 trial, the outcomes of inferior mediastinal lymph node dissection via left thoracotomy versus a transhiatal approach were compared for gastric cancer and EGJ cancers with an esophageal infiltration length ≤ 3 cm. However, the intermediate analysis showed that the incidence of postoperative pneumonia was higher in the left thoracotomy group than in the transhiatal group (13% vs. 4%, *p* = 0.048), and the survival tended to be worse in the left thoracotomy group, so the trial was discontinued. Furthermore, in the final analysis after a long-term follow-up exceeding 10 years, the 5-year survival rate was 37% in the left thoracotomy group but 51% in the transhiatal group, showing a significantly better outcome than the left thoracotomy group.

While the results of the above-mentioned analyses are interesting [9], the subjects were limited to those with a tumor diameter of < 4 cm, And perhaps, the algorithm of dissecting range created based on this analysis will be applied less than half of patients with EGJ cancer. In actual clinical situations, the majority of tumors are considered to have a diameter ≥ 4 cm or an esophageal infiltration length ≥ 3 cm [10, 11]. Indeed, 66.6% of the patients in our study had a tumor size ≥4 cm. As shown in Fig. 1, it is difficult to identify an anatomically accurate EGJ in the case of huge EGJ cancer, and as a result it is impossible to confirm the exact esophageal invasion distance of the tumor (Fig. 1). Kurokawa et al. collected data on Sievert type II tumors from 7 centers and reported on 315 cases [10]. They conducted a multivariate analysis and investigated four variables of histological type (tumor diameter [≥5 cm], pathological T factor [T3 or T4], and esophageal invasion length [≥3 cm]) as factors related to mediastinal lymph node recurrence. However, only the esophageal invasion length was found to be a significant factor. However, in their report, cases from the old age of 1986 were collected, and furthermore, cases with huge tumors exceeding 10 cm were excluded. So, it is presumed that it is difficult to conclude the relation between EGJ cancers and lymph node recurrence form only by their report. Indeed, 5 of our patients had huge tumors exceeding 10 cm in diameter in our study.

Our findings suggest that, for EGJ cancers without a long diameter (< 4 cm), mediastinal dissection might be necessary only for the lower mediastinum, similar to the approach for gastric cancer treatment according to the Japanese guidelines (ver. 5). In contrast, EGJ cancers with a tumor size ≥4 cm may need to undergo thorough surgery with prophylactic lymph node dissection including the upper mediastinum.

In this study, the tumor diameter was measured on the specimen after resection. It is well known that specimens shrink after resection. Therefore, we examined the tumor diameter obtained from preoperative imaging, but in this cohort, there was not a significant difference between the tumor diameter obtained from preoperative imaging and the tumor diameter measured after resection. (Average tumor size: 55.8 mm vs 54.9 mm). In addition, only one case was changed from S group to L group, but it did not significantly affect the overall analysis.

The limitation of this study is that it is a retrospective study targeting a few cases at a single institution. In order to determine the extent of lymph node dissection based on tumor diameter, which is the purpose of this study, it is considered that proof by multi-institutional prospective randomized trial is necessary.

## Conclusion

**Fig. 4:**
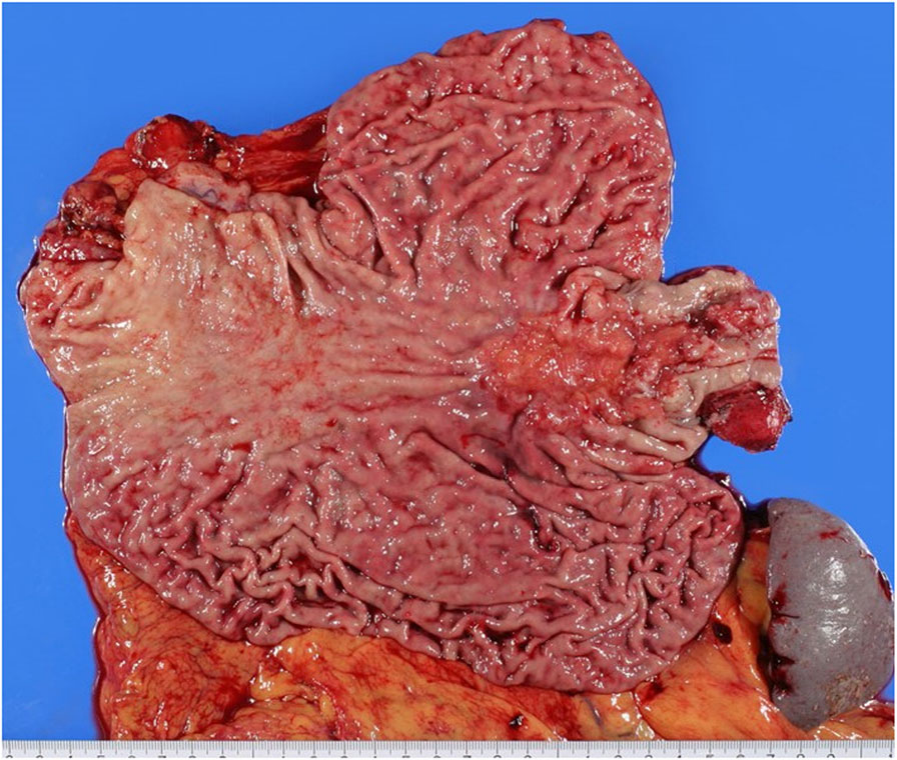
An example of EGJ cancer with a large tumor exceeding 10 cm. In such a case, it is considered difficult to accurately grasp the position of EGJ before surgery

To our knowledge, this is the first report to describe the relationship between the tumor size and lymph node dissection areas. As the tumor of EGJ develops and proliferates, the frequency of lymph node metastasis to the mediastinum seems to increase. Furthermore, as the tumor grows large, it is difficult to accurately judge EGJ on the image, and as a result it is difficult to understand the exact esophageal invasion distance of the tumor (Fig. 4). Therefore, in cases of large tumors involving the EGJ, lymph node dissection including the upper mediastinum is considered vital, regardless of the degree of esophageal invasion.

## Data Availability

The datasets used and/or analyzed during the present study are available from the corresponding author on reasonable request.

## Abbreviations

EGJ: esophagogastric junction
OS: overall survival
RFS: recurrence-free survival

## References

[1] Hongo M, Nagasaki Y, Shoji T (2009). Epidemiology of esophageal cancer: orient to occident. Effects of chronology, geography and ethnicity. J Gastroenterol Hepatol.

[2] Tachimori Y, Ozawa S, Numasaki H, Ishihara R, Matsubara H, Muro K (2018). Comprehensive registry of esophageal Cancer in Japan, 2011. Esophagus: official journal of the Japan Esophageal Society.

[3] Nagy P, Johansson S, Molloy-Bland M (2016). Systematic review of time trends in the prevalence of helicobacter pylori infection in China and the USA. Gut pathogens.

[4] Shiota S, Murakawi K, Suzuki R, Fujioka T, Yamaoka Y (2013). Helicobacter pylori infection in Japan. Expert review of gastroenterology & hepatology.

[5] Azuma N, Endo T, Arimura Y, Motoya S, Itoh F, Hinoda Y (2000). Prevalence of Barrett's esophagus and expression of mucin antigens detected by a panel of monoclonal antibodies in Barrett's esophagus and esophageal adenocarcinoma in Japan. J Gastroenterol.

[6] Eloubeidi MA, Provenzale D (2001). Clinical and demographic predictors of Barrett's esophagus among patients with gastroesophageal reflux disease: a multivariable analysis in veterans. J Clin Gastroenterol.

[7] Westhoff B, Brotze S, Weston A, McElhinney C, Cherian R, Mayo MS (2005). The frequency of Barrett's esophagus in high-risk patients with chronic GERD. Gastrointest Endosc.

[8] Abrams JA, Fields S, Lightdale CJ, Neugut AI (2008). Racial and ethnic disparities in the prevalence of Barrett's esophagus among patients who undergo upper endoscopy. Clinical gastroenterology and hepatology: the official clinical practice journal of the American Gastroenterological Association.

[9] Yamashita H, Seto Y, Sano T, Makuuchi H, Ando N, Sasako M (2017). Results of a nation-wide retrospective study of lymphadenectomy for esophagogastric junction carcinoma. Gastric cancer: official journal of the International Gastric Cancer Association and the Japanese Gastric Cancer Association.

[10] Kurokawa Y, Hiki N, Yoshikawa T, Kishi K, Ito Y, Ohi M (2015). Mediastinal lymph node metastasis and recurrence in adenocarcinoma of the esophagogastric junction. Surgery..

[11] Sasako M, Sano T, Yamamoto S, Sairenji M, Arai K, Kinoshita T (2006). Left thoracoabdominal approach versus abdominal-transhiatal approach for gastric cancer of the cardia or subcardia: a randomised controlled trial. The Lancet Oncology.

[12] Japanese classification of gastric carcinoma: 3rd English edition. Gastric cancer : official journal of the International Gastric Cancer Association and the Japanese Gastric Cancer Association. 2011;14(2):101–12. 10.1007/s10120-011-0041-5 21573743

[13] Japanese Classification of Esophageal Cancer, 11th Edition: part I. Esophagus : official journal of the Japan Esophageal Society. 2017;14(1):1–36. 10.1007/s10388-016-0551-7 PMC5222932 28111535

[14] Japanese Classification of Esophageal Cancer, 11th Edition: part II and III. Esophagus : official journal of the Japan Esophageal Society. 2017;14(1):37–65. 10.1007/s10388-016-0556-2 PMC5222925 28111536

[15] Straatman J, van der Wielen N, Cuesta MA, Daams F, Roig Garcia J, Bonavina L (2017). Minimally invasive versus open esophageal resection: three-year follow-up of the previously reported randomized controlled trial: the TIME trial. Ann Surg.

[16] Biere SS, van Berge Henegouwen MI, Maas KW, Bonavina L, Rosman C, Garcia JR (2012). Minimally invasive versus open oesophagectomy for patients with oesophageal cancer: a multicentre, open-label, randomised controlled trial. Lancet (London, England).

[17] Briez N, Piessen G, Bonnetain F, Brigand C, Carrere N, Collet D (2011). Open versus laparoscopically-assisted oesophagectomy for cancer: a multicentre randomised controlled phase III trial - the MIRO trial. BMC Cancer.

[18] Sihag S, Kosinski AS, Gaissert HA, Wright CD, Schipper PH (2016). Minimally invasive versus open Esophagectomy for esophageal Cancer: a comparison of early surgical outcomes from the Society of Thoracic Surgeons National Database. Ann Thorac Surg.

[19] Mamidanna R, Bottle A, Aylin P, Faiz O, Hanna GB (2012). Short-term outcomes following open versus minimally invasive esophagectomy for cancer in England: a population-based national study. Ann Surg.

[20] Takeuchi H, Miyata H, Gotoh M, Kitagawa Y, Baba H, Kimura W (2014). A risk model for esophagectomy using data of 5354 patients included in a Japanese nationwide web-based database. Ann Surg.

[21] Hulscher JB, van Sandick JW, de Boer AG, Wijnhoven BP, Tijssen JG, Fockens P (2002). Extended transthoracic resection compared with limited transhiatal resection for adenocarcinoma of the esophagus. N Engl J Med.

